# Genetic testing for inherited arrhythmia syndromes and cardiomyopathies: results of the European Heart Rhythm Association survey

**DOI:** 10.1093/europace/euae216

**Published:** 2024-08-16

**Authors:** Ivan Zeljkovic, Anaïs Gauthey, Martin Manninger, Katarzyna Malaczynska-Rajpold, Jacob Tfelt-Hansen, Lia Crotti, Elijah R Behr, Federico Migliore, Arthur Wilde, Julian Chun, Giulio Conte

**Affiliations:** Cardiology Department, Dubrava University Hospital, Zagreb, Croatia; Heart Rhythm Management Center, Universitair Ziekenhuis Brussel-Vrije Universiteit, Brussels, Belgium; Division of Cardiology, Department of Internal Medicine, Medical University of Graz, Graz, Austria; Cardiology Department, Royal Brompton Hospital, Guy’s and St Thomas NHS Foundation Trust, London, UK; Department of Cardiology, Rigshospitalet, Copenhagen University Hospital, Copenhagen, Denmark; Section of Forensic Genetics, Department of Forensic Medicine, Faculty of Health and Medical Sciences, University of Copenhagen, Copenhagen, Denmark; Center for Cardiac Arrhythmias of Genetic Origin, Cardiomyopathy Unit and Laboratory of Cardiovascular Genetics, Department of Cardiology, Istituto Auxologico Italiano, IRCCS, Milan, Italy; Department of Medicine and Surgery, University of Milano-Bicocca, Milan, Italy; Cardiovascular and Genomics Research Institute City St. George’s, University of London and St. George’s University Hospitals NHS Foundation Trust, London, UK; Department of Cardiac, Thoracic, Vascular Sciences and Public Health, University of Padova, Padova, Italy; Department of Clinical and Experimental Cardiology, Heart Center, Amsterdam UMC, Location Academic Medical Center, Amsterdam, The Netherlands; Cardioangiologisches Centrum Bethanien, Agaplesion Bethanien Krankenhaus, Frankfurt, Germany; Division of Cardiology, Cardiocentro Ticino Institute, Ente Ospedaliero Cantonale, via Tesserete 48, 6900 Lugano, Switzerland; Faculty of Biomedical Sciences, USI, via la Santa 1, 6900 Lugano, Switzerland

**Keywords:** Sudden cardiac death, Inherited arrhythmogenic diseases, Inherited primary arrhythmia syndromes, Cardiomyopathies, Genetic heart disease, Genetic testing, EHRA survey

## Abstract

**Aims:**

Indications and clinical impact of genetic testing for cardiac diseases have increased significantly over the past years. The aim of this physician-based European Heart Rhythm Association (EHRA) survey was to assess current clinical practice and access to genetic testing for cardiac diseases across European Society of Cardiology countries and to evaluate adherence to the 2022 EHRA/HRS/APHRS/LAHRS Expert Consensus Statement on genetic testing.

**Methods and results:**

An online questionnaire composed of 28 questions was submitted to the EHRA Research Network and European Reference Network GUARD-Heart healthcare partners and promoted via dedicated social media channels. There were 357 respondents from 69 countries, 40% working in a hospital setting with a cardiac genetic service and/or a dedicated clinic focusing on inherited cardiac diseases and 27% with an onsite genetic laboratory. No genetic testing or low annual rate (<10/year) was declared by 39% of respondents. The majority of respondents (78%) declared issues or limitations to genetic testing access in their clinical practice. The main reasons for not providing or limited access to genetic testing were no availability of dedicated unit or genetic laboratory (35%) or reimbursement issues (25%). The most frequently reported indication for genetic testing was diagnostic purpose (55%). Most respondents (92%) declared offering genetic testing preceded by genetic counselling and 42% regular multidisciplinary evaluations for patients with cardiac genetic diseases. The perceived value of genetic testing in the diagnostic, prognostic, and therapeutic assessment was variable (67, 39, and 29%, respectively) and primarily based on the specific inherited disease. The majority of respondents recommended cascade genetic testing for the first-degree family members in case of pathogenic/likely pathogenic variant in the proband.

**Conclusion:**

This survey highlights a significant heterogeneity of genetic testing access and provision and issues attributable to the availability of dedicated unit/genetic laboratory and reimbursement. However, adequate adherence to indications in the current recommendations for genetic testing in patients with cardiac diseases was observed.

## Graphical Abstract

**Graphical Abstract:**
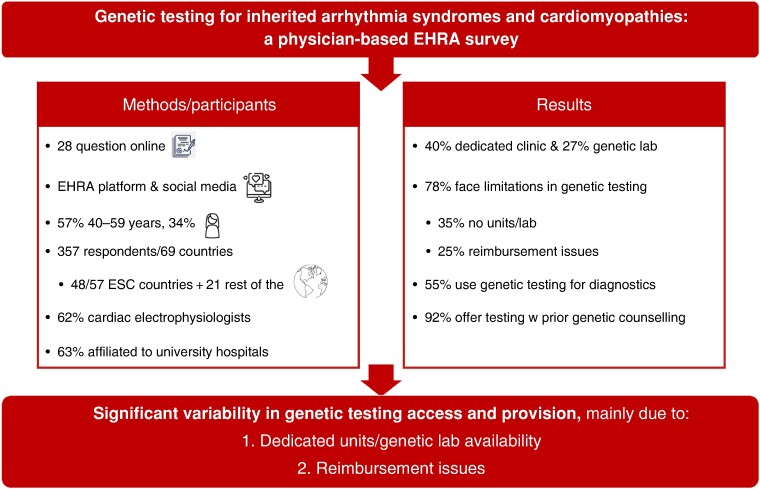

## Introduction

Inherited primary arrhythmia syndromes and cardiomyopathies are two groups of cardiac genetic diseases associated with an increased risk of sudden cardiac death (SCD) and/or heart failure.^1–4^ The diagnostic approach to these diseases has been reported to be highly heterogeneous across European centres, with underuse of genetic testing more likely to occur in centres without dedicated units on channelopathies/cardiomyopathies.^5,6^

Indications and clinical impact of genetic testing for cardiac diseases have increased significantly over the past years. Recently, an Expert Consensus Statement on the state of genetic testing for cardiac diseases was issued by the European Heart Rhythm Association (EHRA) in collaboration with international cardiac societies.^7^ The document presented the state of genetic testing for inherited arrhythmia syndromes, cardiomyopathies, and SCD, shedding light on the diagnostic, prognostic, and therapeutic implications of genetic testing in these diseases.

Despite its established clinical value in terms of more diagnostic precision and influence on therapeutic options and prognosis, the feasibility and access to genetic testing may be limited not only by logistical barriers and the absence of dedicated professionals but also by costs and reimbursement policies.^7,8^ The aim of this physician-based EHRA survey was to assess current clinical practice and access to genetic testing for cardiac channelopathies and cardiomyopathies across European Society of Cardiology (ESC) countries and to evaluate adherence to the 2022 EHRA/HRS/APHRS/LAHRS Expert Consensus Statement.

## Methods

This physician-based survey was developed and disseminated by EHRA in collaboration between the Scientific Initiatives Committee (SIC), the Young Electrophysiologists (YEP) Committee, the ECGen Focus Group of EHRA, and the European Reference Network for rare cardiac diseases, GUARD-Heart. An online 28-item questionnaire was developed and circulated to the EHRA Research Network, ECGen members, GUARD-Heart healthcare partners, and dedicated social media channels between 6 October and 5 December 2023.

The physician-based survey was constructed to collect information regarding current clinical usage of cardiac genetic testing and adherence to recommendations, focusing on the following inherited diseases: long QT syndrome (LQTS), Brugada syndrome (BrS), catecholaminergic polymorphic ventricular tachycardia (CPVT), short QT syndrome (SQTS), idiopathic ventricular fibrillation (IVF)/unexplained sudden cardiac arrest (SCA), early repolarization syndrome (ERS), progressive cardiac conduction defect (PCCD), arrhythmic mitral valve prolapse and dilated, hypertrophic, arrhythmogenic, left ventricular non-compaction (LVNC), and restrictive cardiomyopathies.

The online-based questionnaire consisted of single- and multiple-choice questions assessing physicians’ daily practice on cardiac genetic testing, its availability, indications, reimbursement, and compliance with new recommendations. The results of the anonymized data about participants, their institutions, and services were also collected in compliance with the European General Data Protection Regulation (GDPR) 2016/679. Survey results are expressed as categorical data (numbers and proportions). The statistical analysis was performed using SPSS Version 20 (IBM SPSS Statistics, New York, USA).

## Results

A total of 357 respondents from 69 countries participated in the questionnaire. The mean age of the respondents was 47 ± 6 years, and 34% (*N* = 121) were females. Forty-eight (84%) of the 57 ESC National Cardiac Societies were represented in the survey, with the addition of 21 non-ESC countries represented with at least one participant. The most represented country was Croatia (16%), followed by Italy (13%) and Belgium (7%).

Of the respondents, 27% (*N* = 98) were general cardiologists, 62% (*N* = 223) had specific competencies in cardiac electrophysiology, 12% (*N* = 44) in cardiogenetics, 13% (*N* = 48) in heart failure, 11.5% in cardiac imaging (*N* = 41), and 6% (*N* = 22) in paediatric cardiology.

### Institutional setting

Respondents were affiliated with university hospitals (*N* = 225, 63%), non-university hospitals (*N* = 82, 23%), private hospitals (*N* = 29, 8%), or private practice (*N* = 21, 6%).

**Figure 1:**
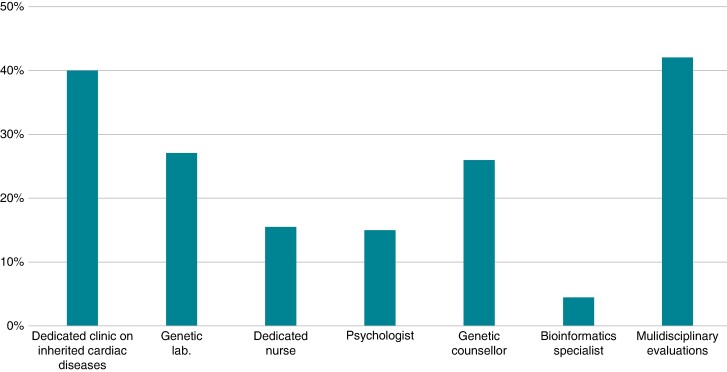
Institutional setting and dedicated facilities/personnel.

Approximately 40% (*N* = 143) of the respondents declared the presence at their institution of a dedicated clinic on inherited cardiac diseases or a cardiac genetic service, and 27% (*N* = 96) presence of a genetic laboratory. Presence of an institutional dedicated nurse was declared by 55 respondents (15.4%), psychologist by 54 (15%), genetic counsellor by 93 (26%), and bioinformatics specialist by 20 (5.6%) (*Figure 1*).

Most respondents (*N* = 328, 92%) declared offering genetic testing preceded by genetic counselling performed by a cardiologist (*N* = 206, 62.8%) or by a geneticist/genetic counsellor (122, 37.2%). The main reason for not providing genetic counselling was the lack of a dedicated specialist at the institution.

Regular multidisciplinary evaluations for patients with cardiac genetic diseases were reported by 42% (*N* = 151) of the respondents and included the involvement of geneticists (*N* = 130, 86%), pathologists (*N* = 57, 38%), and paediatric cardiologists (*N* = 118, 78%).

### Current status of genetic testing for cardiac diseases

**Figure 2:**
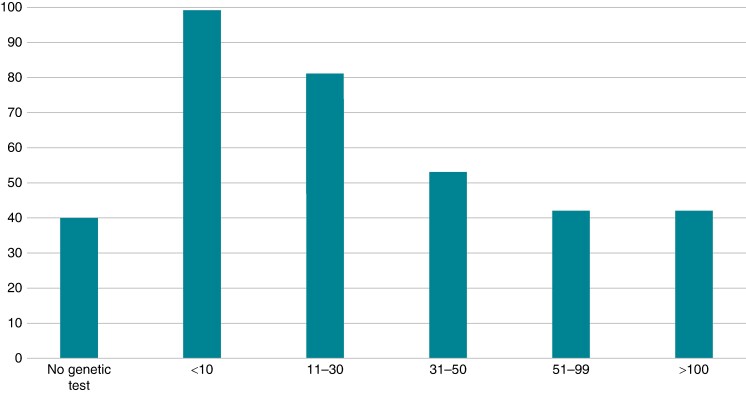
Number of genetic tests in the last 12 months.

The mean number of genetic tests performed per centre in the last year was 35 ± 11. There were 40 respondents (11.2%) declaring no genetic test in the last year, 99 (27.7%) declaring <10 genetic test, and 43 > 100 genetic test (12%) (*Figure 2*).

Ninety-five respondents (30%) sent the samples to a regional genetic laboratory, 86 (27%) to a national specialized genetic laboratory, and 70 (22%) abroad to an international centre. The remaining 66 (21%) used the institutional genetic laboratory for cardiogenetic testing. The main reason for requesting a genetic testing to an international centre was the lack of a local or regional genetic laboratory (68%) and/or absence of dedicated units and counselling (32%).

**Figure 3:**
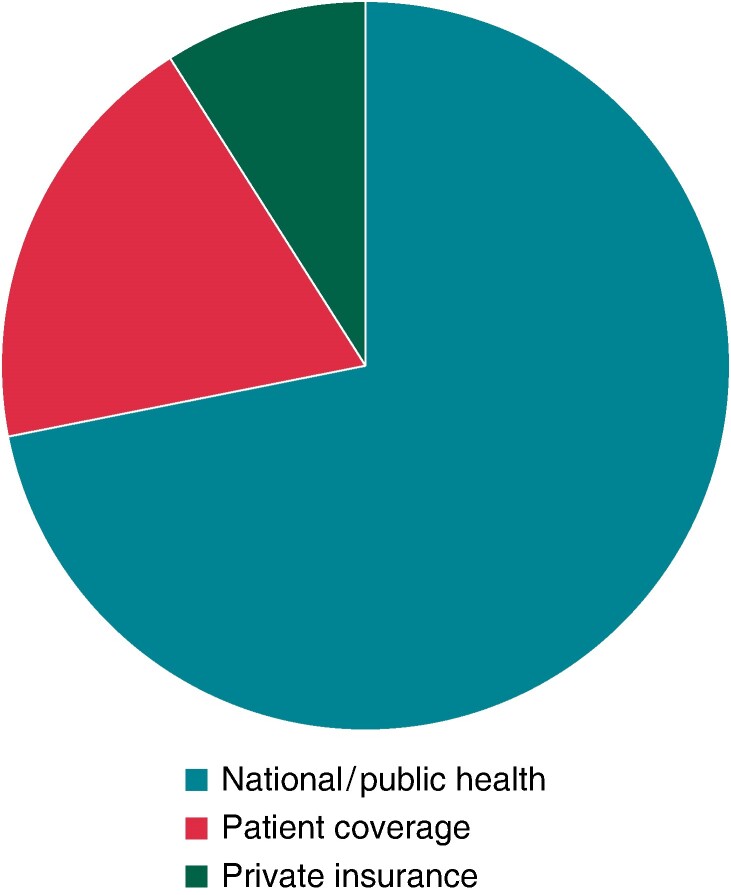
Genetic testing coverage.

The request for genetic testing for cardiac disease by a cardiologist was declared allowed by the majority of respondents (79%), while 21% declared the necessity of the request by a geneticist. Regarding genetic testing, panel sequencing was the most commonly requested test in the last year (119/317, 37.5%). There were 198 respondents (62.5%) not aware of the specific adopted sequencing technique. Twenty-one (6.6%) and 17 (5.3%) declared, respectively, the possibility of performing whole-exome sequencing (WES) or whole-genome sequencing (WGS) in specific cases or for research purposes. More than half of respondents (194, 54%) declared that genetic testing was mainly reimbursed by national/public health funds in almost all cases and 64 (18%) upon indication review and approval by an institutional committee. Routine genetic testing coverage by the patient was reported by 68 (19%) and by private funds by 31 (9%) of respondents (*Figure 3*).

### Indications

**Figure 4:**
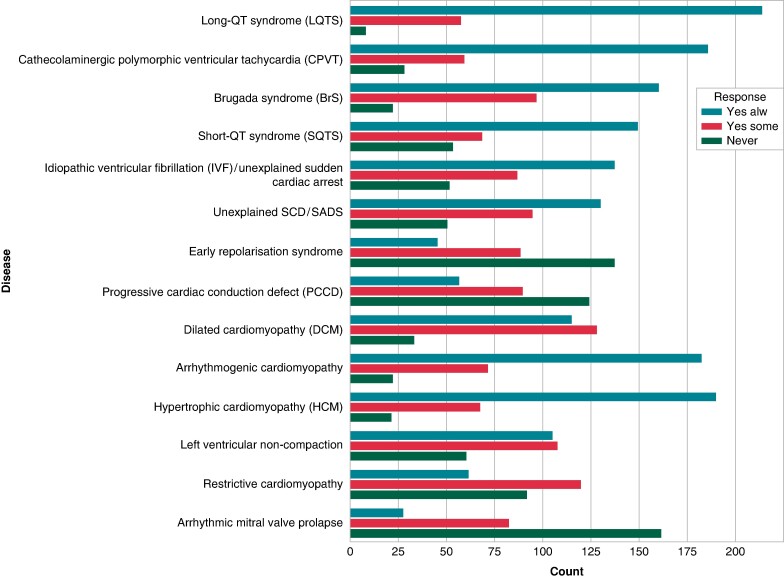
Diagnostic assessment by genetic testing of specific cardiac genetic diseases.

**Figure 5:**
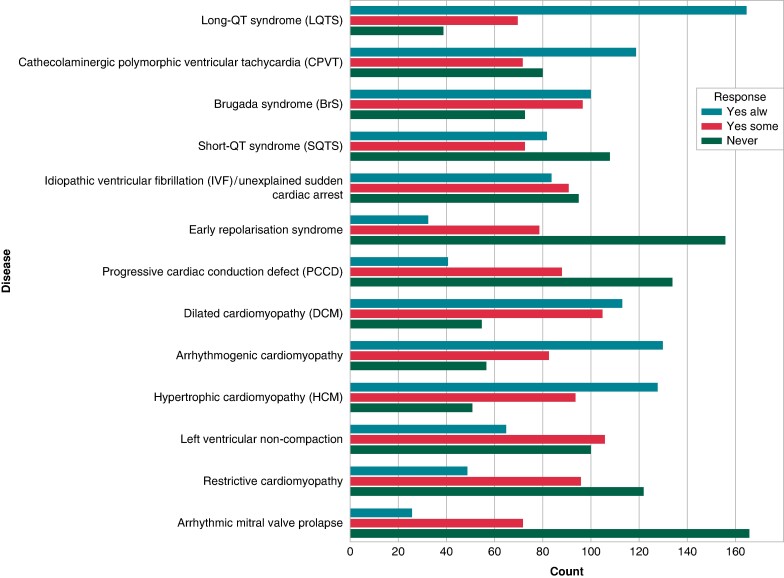
Prognostic assessment by genetic testing of specific cardiac genetic diseases.

Genetic testing was most frequently required for diagnostic purposes (213, 67.2%). One-hundred and twenty-four (39%) and 91 (28.7%) respondents declared, respectively, genetic testing to evaluate prognostic and therapeutic implications alongside diagnostic aspects. Clinical usage of genetic testing in the diagnostic and prognostic assessment of specific inherited cardiac diseases is depicted in *Figures 4* and *5*.

Among channelopathies, the disease most frequently assessed with genetic testing for diagnostic purposes was LQTS, followed by BrS and CPVT. Among cardiomyopathies, arrhythmogenic cardiomyopathy was the most commonly assessed disease, followed by hypertrophic and dilated cardiomyopathy (*Figure 4*). Regarding prognostic assessment, the most frequently examined diseases were LQTS, CPVT, and BrS among channelopathies and arrhythmogenic, hypertrophic, and dilated cardiomyopathies (*Figure 5*).

In case of a confirmed pathogenic/likely pathogenic (P/LP) variant in a proband, clinical screening with cascade genetic testing was recommended for the first-degree family members by 200 respondents (63%). Most of these respondents (150/200, 75%) indicated performing predictive genetic testing of P/LP variants in children. Most of them did not declare any specific age cut-off for testing and based the temporal decision according to the specific disease.

Nearly 67% of the respondents (213/317) did not perform a co-segregation analysis of variants of unknown/uncertain significance (VUS) to assess variant pathogenicity.

One-hundred and eighty-one respondents (57%) declared offering VUS reassessment over time. Half of them (53%) reported no specific reassessment temporal strategy for VUS, while 9 and 29% reported VUS reassessment every 2 years and between 2 and 5 years, respectively.

Finally, 88% of the respondents consider the ‘2022 EHRA/HRS/APHRS/LAHRS Expert Consensus Statement on the state of genetic testing for cardiac diseases’ valuable for their current clinical practice.

### Issues and barriers to genetic testing

The majority of respondents offering genetic testing (247, 78%) declared having encountered issues or limitations to access the genetic testing in their clinical practice. The main reasons for not providing or limiting access to genetic testing were no availability of a dedicated cardiogenetic service or genetic lab (35%) and reimbursement issues (25%), followed by the absence of genetic counselling in the centre (17%).

**Figure 6:**
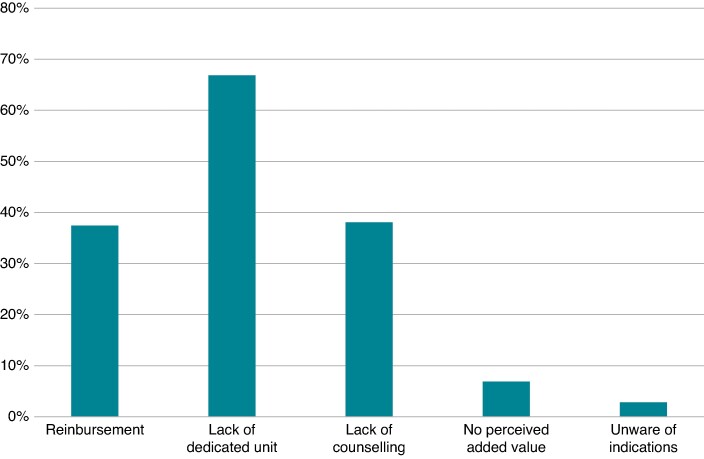
Reasons for not providing genetic testing or for limited access (<10/year).

Of 139 respondents declaring no or limited number of genetic tests (<10/year), issues related to reimbursement were reported by 52 (37.4%), a lack of dedicated units by 93 (66.9%), and absence of proper counselling by 53 (38%). The perception that genetic testing does not add value to prognostic and/or therapeutic clinical course/decisions was reported by 10 respondents (7.2%). In contrast, only a minority (4, 2.9%) declared being unaware of the specific indications for genetic testing (*Figure 6*).

## Discussion

This report highlights different important features of current practice on genetic testing for cardiac diseases: (i) one out of three respondents declared having requested no or <10 genetic tests in the last 12 months; (ii) issues to genetic access and provision are commonly experienced and are mostly related to the absence of dedicated units on cardiac diseases or cardiogenetic services; and (iii) the perceived value of genetic testing in the diagnostic and prognostic assessment is variable. However, adequate adherence to current guidelines and expert consensus statements in terms of indications, counselling, and cascade screening is observed.

### Institutional setting for genetic cardiac disease

Nearly 60% of respondents in this survey declared no dedicated clinic or genetic service at their institution, and 73% indicated absence of a genetic laboratory on site. Interestingly, 22% of the respondents request genetic testing that is performed abroad.

The 2022 expert consensus document states that genetic testing in patients with a potential cardiogenetic condition requires appropriate genetic counselling.^7^ In line with this statement, the vast majority of respondents of this survey declared offering genetic testing preceded by genetic counselling. Conversely, regular multidisciplinary evaluations were reported only by a suboptimal rate (42%) of respondents. Indeed, it is established that variant interpretation in the clinical setting is greatly enhanced by the use of disease-specific, multidisciplinary teams that could include clinical disease experts, clinical geneticists, genetic counsellors, and molecular geneticists.^7^

Regarding sequencing strategy, in addition to single-gene testing and gene panel testing, there is now the ability to perform WES and WGS. However, these sequencing techniques are reported only by a minority of participants (7 and 5%, respectively), and panel sequencing remains the most commonly adopted sequencing strategy (38%). Interestingly, there was a significant number of respondents (62%) not aware of the adopted sequencing technique. In patients with a clear specific phenotype, it is appropriate to perform genetic testing and analyse genes with definite or strong supporting evidence. Broader genetic testing may be considered in selected cases with a definite phenotype and no genetic diagnosis after testing the genes with definite or strong evidence supporting disease causation.^7^

### Indications and perceived value of genetic testing

The diagnostic, prognostic, and therapeutic impact of genetic testing for the proband relies on the specific genetic disease. In line with the consensus statement document, genetic testing for diagnostic assessment was frequently reported for patients with LQTS, CPVT, dilated, hypertrophic, and arrhythmogenic cardiomyopathies. A considerable number of respondents considered genetic testing valuable for the diagnosis of BrS.

The use of genetic testing has become evident for risk stratification and for enhancing precision medicine approaches and therapeutic strategies.^4^ Accordingly, 39 and 29% of respondents reported genetic testing for risk stratification and therapeutic choices, mostly for patients with LQTS, CPVT, BrS, and dilated, arrhythmogenic, and hypertrophic cardiomyopathies.

The opinion that genetic testing does not add any value or not being aware of any specific indication was a reason for not providing genetic testing only in a minority of respondents, proving high acknowledgement and adherence to the current guidelines and recommendations.

In the 2011 EHRA/HRS Expert Consensus Statement, genetic testing was recommended for probands with a clinical diagnosis and all family members of a successfully genotyped proband (class I recommendation).^9^ The 2022 EHRA/HRS/APHRS/LAHRS Expert Consensus Document indicates that in families where a P/LP variant has been identified, detailed genetic counselling and guidance regarding inheritance patterns, variant penetrance, and risk should be offered, and cascade testing facilitated.^7^ In line with the two documents, most respondents reported cascade testing in families with P/LP variants even in adult and paediatric patients. However, most of them did not declare any specific temporal strategy for testing and based their decision according to the specific disease and its clinical manifestation.

### Barriers to genetic testing

The results of this survey strengthen previously reported findings on the limited use of genetic testing for patients with cardiac diseases in daily practice. In a previous EHRA centre-based survey on the management of patients with inherited arrhythmia syndromes, centres without a dedicated unit performed less genetic testing for all the different types of channelopathies, including those where a genetic diagnosis could influence therapeutic choices.^6^ In this physician-based survey, including channelopathies and all types of cardiomyopathies, no genetic testing or a low annual rate was reported by a considerable number of respondents (39%). The most commonly reported reasons for limited genetic testing was the lack of dedicated units/professionals and reimbursement issues.

The creation and implementation of dedicated units, where patients and their families are seen in a multidisciplinary setting by dedicated professionals, is of utmost importance for ensuring a proper management of patients with genetic cardiac diseases.

Scientific international societies can play an active and important role in enhancing the promulgation and improved uptake of evidence-based management recommendations for genetic testing in patients with cardiac diseases and ensure homogenous provision across all ESC countries. Genetic testing could in the future become a quality indicator for healthcare providers.^10^ Further efforts should also be carried out to overcome reimbursement policy issues.

### Limitations

This survey has different limitations. Due to the relatively limited number of respondents, mainly electrophysiologists affiliated with university hospitals, and especially unequal representation among countries, the results cannot be extrapolated to different categories of practitioners and all ESC and European countries. The rate of respondents declaring limited request of genetic testing in the last 12 months may be due to the fact that some of them do not manage patients with genetic cardiac diseases and may not be directly involved in the test request.

## Conclusions

This survey highlights a significant heterogeneity of genetic testing access and provision and issues attributable to the availability of dedicated units/cardiac genetic services and reimbursement. However, adequate adherence to the current recommendations for genetic testing in patients with cardiac diseases about indications, cascade screening, and counselling is observed.
